# Editorial: Food preservation and pretreatment methods for optimal nutrient retention

**DOI:** 10.3389/fnut.2024.1465139

**Published:** 2024-07-31

**Authors:** María Hernández Carrión, César Ozuna, Julieta del Carmen Villalobos Espinosa

**Affiliations:** ^1^Department of Chemical and Food Engineering, Grupo de Diseño de Productos y Procesos (GDPP), Universidad de los Andes, Bogotá, Colombia; ^2^Departamento de Alimentos, División de Ciencias de la Vida, Campus Irapuato-Salamanca, Universidad de Guanajuato, Guanajuato, Mexico; ^3^Tecnológico Nacional de México/ITS de Teziutlán, Ingeniería en Industrias Alimentarias, Teziutlán, Mexico

**Keywords:** food preservation, nutrient retention, alternative technologies, green technologies, emerging technologies

In current times, consumers have become increasingly aware of the role that food plays in their health (1). Consequently, there has been a growing demand for functional, healthy, nutritious, and environmentally sustainable food products (2). This demand has motivated the food industry to seek non-conventional food preservation and pretreatment methods that preserve the organoleptic, technological, and nutritional properties of food products while minimizing environmental impact and reducing waste generation (3).

In addition, it has been shown that the assimilation of some functional and nutritional compounds from food products is relatively low compared to the amount ingested (4). Certain preservation and processing treatments applied to foods can produce structural and chemical modifications that can influence the fraction of nutrients that are released from the food matrix and subsequently absorbed during digestion. These modifications can increase the bioavailability of the compounds of interest (5–7) (Largo-Avila et al.; Williams-Ngegba et al.; Zhang R. et al.).

This Research Topic aims to present and highlight the potential use of innovative and emerging preservation and pretreatment technologies to enhance the nutrient retention of food products. It seeks to compile a collection of original research articles that present the latest advances and innovative solutions in food preservation and pretreatment methods to enhance the quality, nutrient retention, and functionality of food products. The research areas include CO_2_ treatment, sun drying, refractance window drying, holder pasteurization, high-temperature short-time pasteurization, high-pressure processing, UV radiation, pulsed electric fields, acetic acid treatment, and chilled storage.

This Research Topic contains six (6) original articles and one (1) review, which are listed below with a summary of the main results they present:

1. ***Comparison of physicochemical and volatile flavor properties of neon flying squid (Ommastrephes bartramii), jumbo squid (Dosidicus gigas), and Argentine shortfin squid (Illex argentinus) during chilled storage*** by Huo et al.
  This study thoroughly investigates the similarities and differences in the physicochemical properties of neon flying squid, jumbo squid, and Argentine squid mantles during chilled storage and provides important insights for evaluating the performance of different squids during storage.
2. ***Footprint analysis of CO***_**2**_ ***in microbial community succession of raw milk and assessment of its quality*** by Zheng et al.
  The effects of CO_2_ treatment on the succession footprint of the microbial community and changes in quality during the period of raw milk chilling were examined using 16S rRNA analysis combined with electronic nose and electronic tongue techniques. This study provides new theoretical insights into the industrial application of CO_2_ in raw milk.
3. ***Variations in micronutrient concentrations and retentions in fufu made from yellow-fleshed cassava as a function of genotype and processing methods*** by Williams-Ngegba et al.
  This study looked at how the concentration and retention of micronutrients in yellow-fleshed cassava fufu varied depending on genotype and processing method (conventional or traditional). The results showed the modified traditional river method obtained the highest true retention percentage of total β-carotene, while sun-drying was the best method for iron and zinc true retention percentages.
4. ***Effect of Refractance Window™ and oven drying on physicochemical and sensory properties of peach (Prunus persica L.) surplus*** by Largo-Avila et al.

This study compared the effects of different drying methods (conventional oven drying and refractance window drying) on the physical and sensory properties of peach surplus. The findings highlight the potential of refractance window drying on the consumer's acceptability of dried peach products, offering promising opportunities for surplus peach utilization in the food market.

5. ***Breast milk preservation: thermal and non-thermal processes and their effect on microorganism inactivation and the content of bioactive and nutritional compounds*** by Núñez-Delgado et al.
  This review aims to enhance the understanding of preservation techniques for human breast milk (HBM) and provide a comprehensive overview of the current state of HBM treatment. It addresses microbial concerns, focusing on critical pathogens like *Staphylococcus aureus, Enterococcus, Escherichia coli, Listeria monocytogenes*, and Cytomegalovirus; it also explores how various preservation methods such as holder pasteurization, high-temperature short-time pasteurization, high-pressure processing, UV radiation, and pulsed electric fields can mitigate these risks.
6. ***Evaluation of acetic acid treatment of fresh-cut water chestnuts using grey-correlation analysis based on the variation-coefficient weight*** by Zhang Y. et al.
  This study aimed to select the optimal treatment through a comprehensive comparison of the preservation effect of acetic acid, which could prolong the shelf life of fresh-cut water chestnuts and improve their storage quality. The results suggest that acetic acid, a simple, safe, and economically effective preservative, was suitable for preserving fresh-cut water chestnuts. These findings provide information and comprehensive evaluation methods for preserving fresh-cut fruits and vegetables.
7. ***Transcriptome analysis reveals the effect of cold storage time on the expression of genes related to oxidative metabolism in Chinese black truffle*** by Zhang R. et al.
  In this research, both transcriptome and physicochemical analyses were conducted to investigate changes in nutrients and gene expression in truffle fruiting bodies during cold storage. This study provides strong experimental evidence to support the development of truffle preservation technologies aimed at improving the quality of truffles under storage and investigating the molecular and physiological mechanisms underlying the physicochemical variations in *T. indicum* after harvest.

The articles in this Research Topic suggest that in the coming years, the food industry will remain focused on using alternative and innovative preservation and processing technologies that improve nutrient retention of food products, are eco-friendly, and promote environmental sustainability and the circular economy. Additionally, it is important to highlight that, to meet consumer demands, food processing and preservation conditions will largely be influenced by techniques that help maintain food properties and enable the controlled release of beneficial compounds.

## Author contributions

MH: Writing – original draft. CO: Writing – review & editing. JV: Writing – review & editing.
